# Supplementary figures and images for: Dynamic phosphorylation of Hcm1 promotes fitness in chronic stress
Source: PLoS Genet. 2025 Sep 15;21(9):e1011874. doi: 10.1371/journal.pgen.1011874 (PMC12453243; doi:10.1371/journal.pgen.1011874)

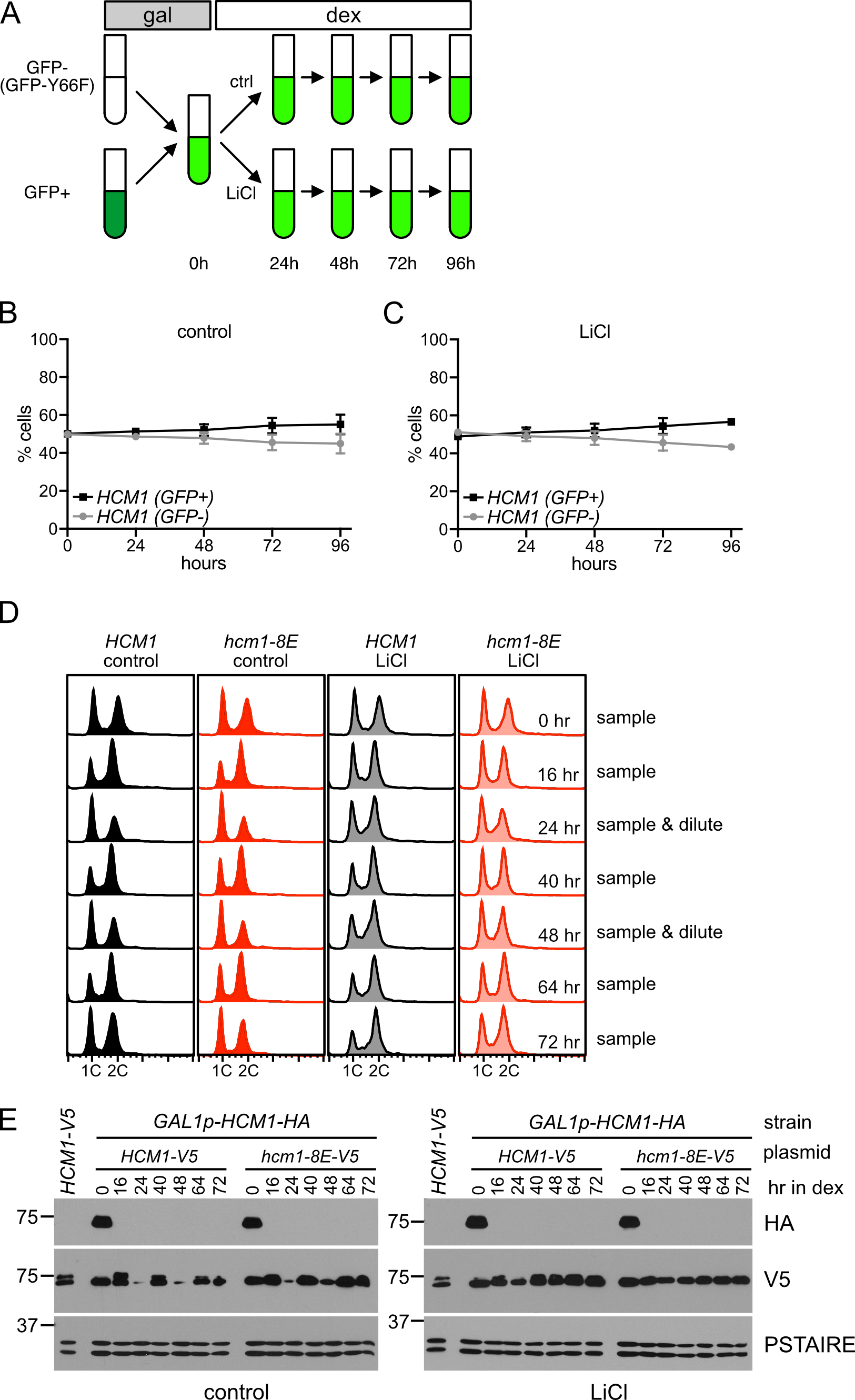

Supplement: S1 Fig — (A) Schematic of pairwise competitive fitness assays. (B-C) GFP markers do not influence fitness. WT (HCM1) strains expressing GFP (GFP+) or GFP-Y66F (GFP-) were co-cultured in control medium (B) or medium containing LiCl (C). Strains have equivalent fitness. (D-E) HCM1 and hcm1-8E strains were grown in monoculture following the dilution protocol used for Phosphosite Scanning. (D) DNA content as measure by flow cytometry, which is used to infer cell cycle position. (E) Western blots showing Hcm1 expression levels. WT Hcm1 that is expressed from the genome under the control of the GAL1 promoted was detected with HA-antibodies and was undetectable after 16 hours of growth in dextrose (dex). Plasmid expressed copies of Hcm1 are tagged with V5 and were expressed throughout the experiment. In control medium, levels of Hcm1 decrease at 24 and 48 hours when cells reach saturation but increase again as cells resume cycling. Hcm1-V5 expressed from the genomic locus is included for comparison, demonstrating that the plasmid expressed copy of Hcm1 is not overexpressed. PSTAIRE is shown as a loading control. (TIFF) [file pgen.1011874.s001.tiff]

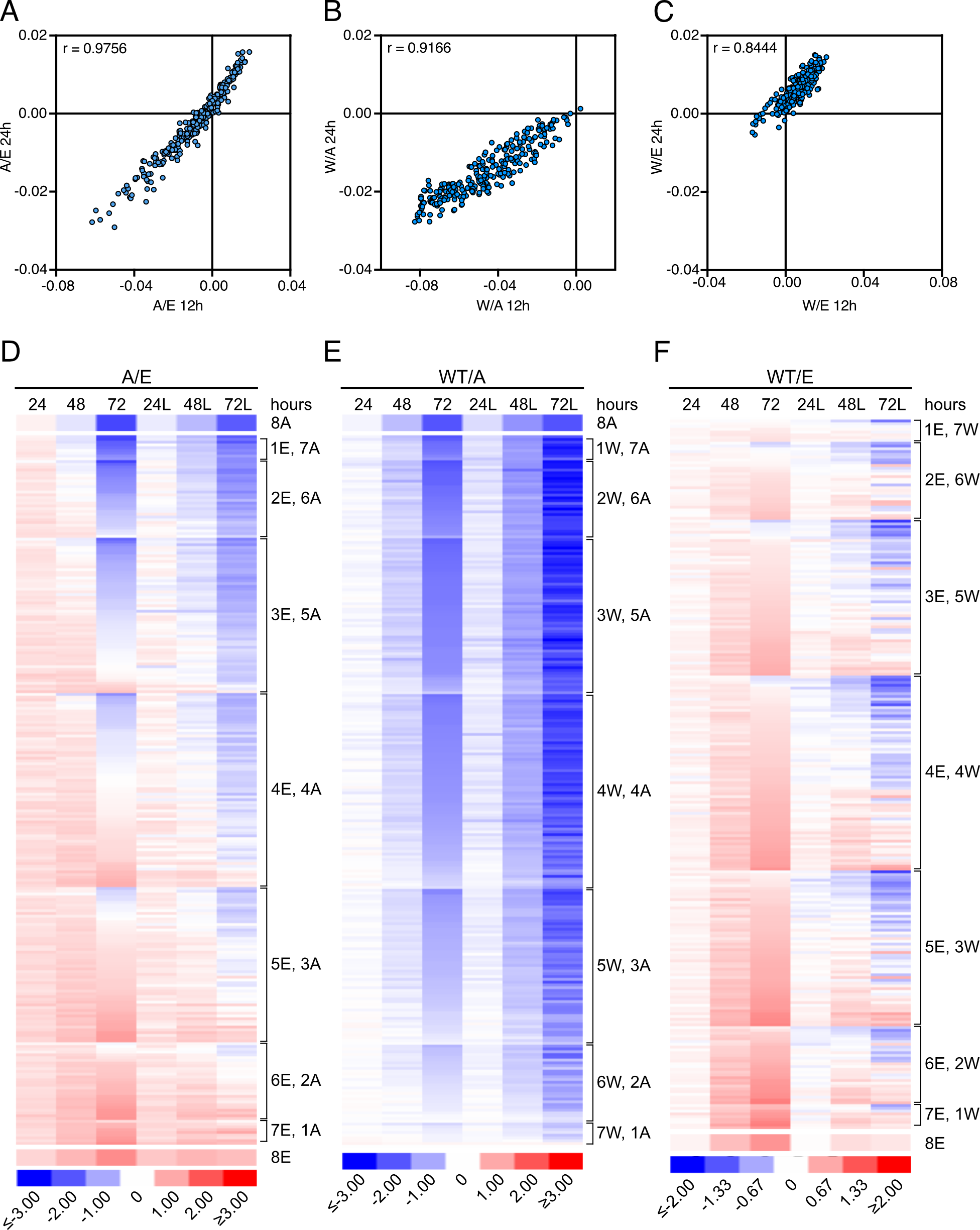

Supplement: S2 Fig — (A-C) Scatter plots comparing selection coefficients derived from Hcm1 Phosphosite Scanning screens carried out with dilutions every 12-hour time points, to maintain logarithmic growth, (from [20]) to those carried out with dilution every 24-hours. (A) shows screening of the Hcm1 A/E library, (B) shows screening of the Hcm1 WT/A library, (C) shows screening of the Hcm1 WT/E library. (D-F) Heat maps showing log2 fold change values of each mutant in Phosphosite Scanning screens performed in control medium or LiCl (L). Each row represents a mutant, shown is the log2 fold change of normalized read counts with respect to time zero for each mutant, all mutants have been normalized to WT. Blue indicates depletion, red indicates enrichment. Mutants are clustered by the number of phosphomimetic mutations and ranked by increasing log2 FC within each cluster in control conditions. Shown is an average of n = 4 biological replicates. (TIFF) [file pgen.1011874.s002.tiff]

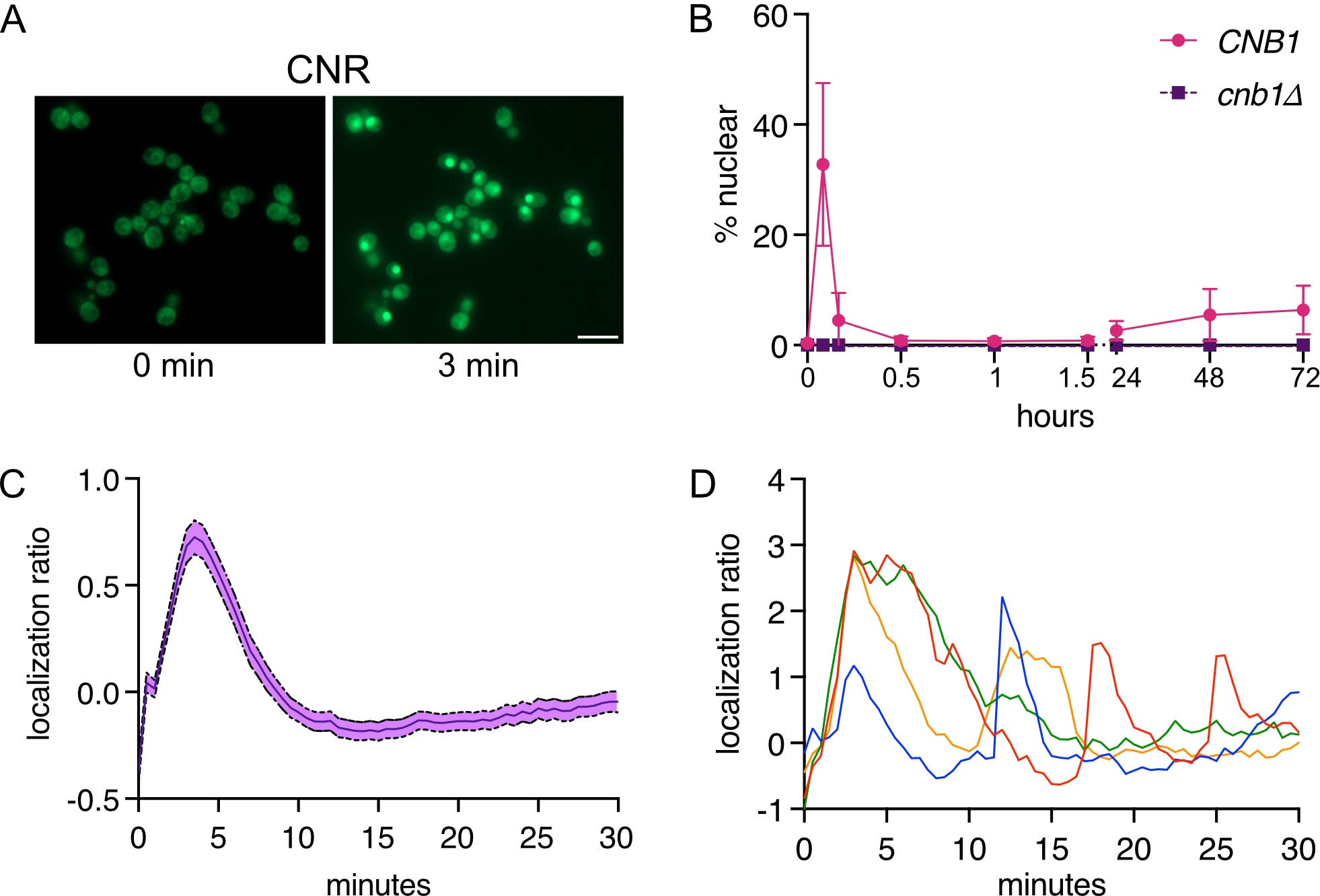

Supplement: S3 Fig — (A) Representative image of cells expressing a GFP-tagged CN-reporter (CNR) that localizes to the nucleus upon CN activation. Cells are shown before and after treatment with 150mM LiCl for 3 minutes. Scale bar represents 10μm. (B) Active CN is detected in WT cells through 72 hours of LiCl treatment. WT (CNB1) and CN-mutant (cnb1Δ) cells expressing CNR were treated with 150mM LiCl and imaged. Shown is the percentage of cells with nuclear-localized reporter at the indicated time points. Data is an average of n = 3 experiments, error bars represent standard deviation. (C) Maximal CN activation peaks at approximately 5 minutes after LiCl addition. WT cells expressing the CN reporter were treated as in (A) and imaged at 30 second intervals for 30 minutes. Average localization ratio from n = 405 cells is shown, shaded area represents 95% confidence interval. (D) Representative traces of 4 cells from experiment in (C) showing short pulses of CN activation occur after the initial nuclear signal diminishes. (TIFF) [file pgen.1011874.s003.tiff]

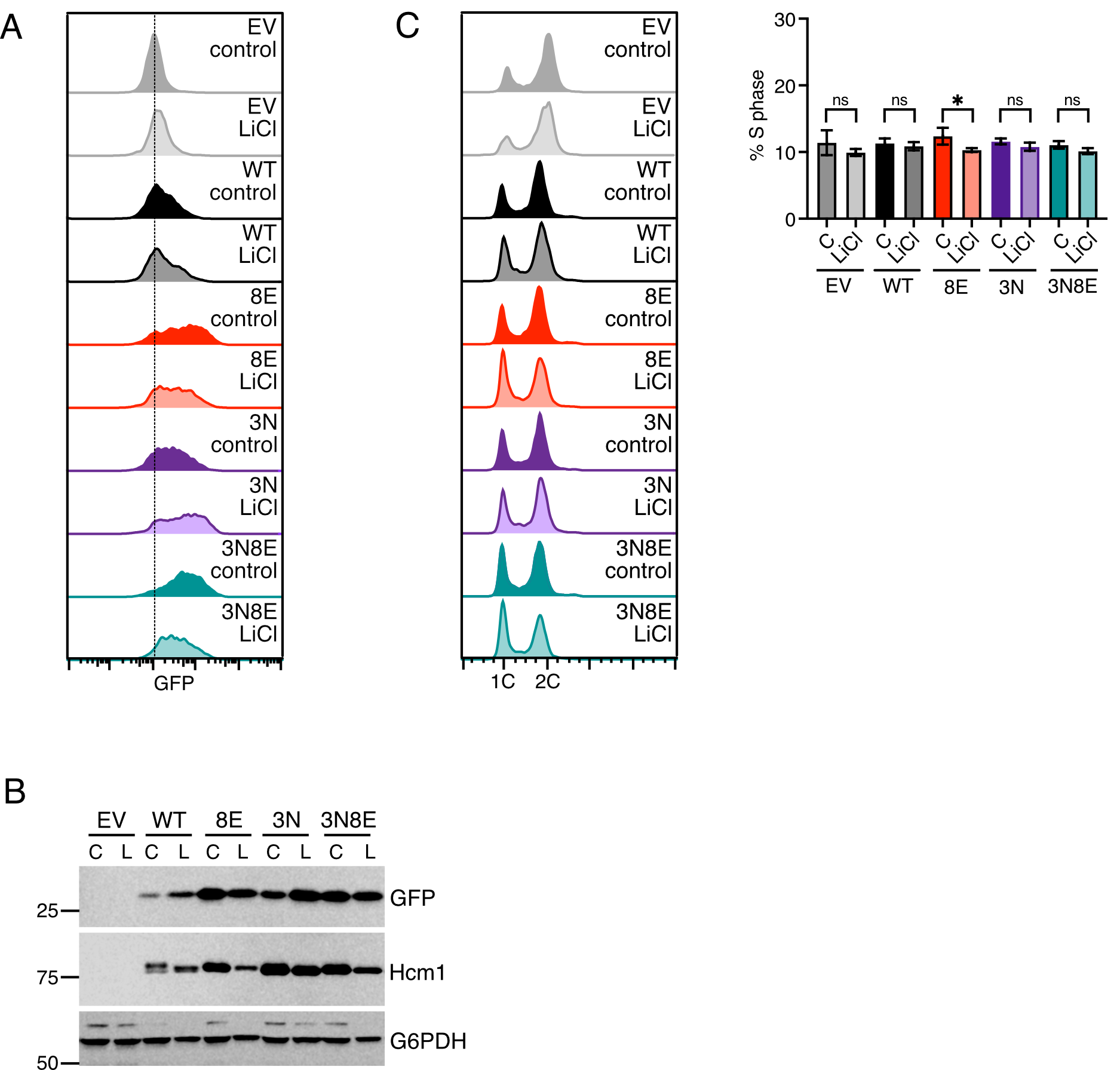

Supplement: S4 Fig — (A) FACS plots showing the distribution of GFP expression levels in cells expressing the indicated Hcm1 mutants or an empty vector (EV), after 40 hours of growth in control medium or medium with LiCl. Shown is a representative experiment from one of n = 6 replicates included in Fig 7A. (B) Western blots showing expression of GFP, Hcm1 mutants, and G6PDH (loading control) from a representative experiment from one of n = 6 included in Fig 7A. (C) FACS analysis of mutants included in Fig 7A after 40 hours of growth in control of LiCl medium. Representative plots from one of n = 6 experiments are shown on the left. A graph of the average percentage of cells in S-phase from all replicates is shown at the right. Error bars represent standard deviation. Significance was calculated comparing the percent S-phase in cultures in control vs LiCl medium using two-way ANOVA with Šídák’s multiple comparisons test was used to test significance, *p < 0.01. (TIFF) [file pgen.1011874.s004.tiff]

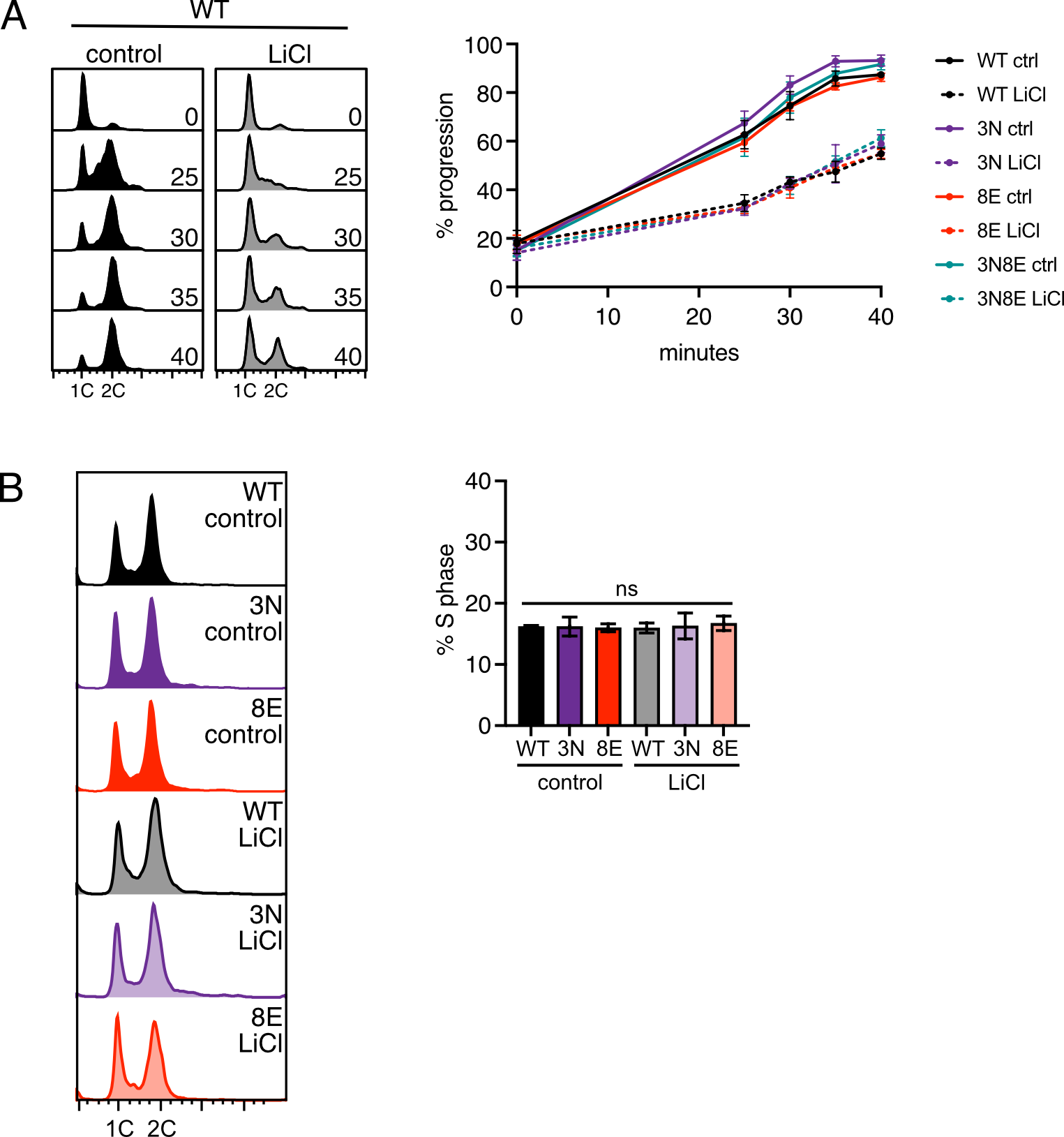

Supplement: S5 Fig — (A) WT and Hcm1 mutant cells do not release synchronously from a G1 arrest after they have adapted to LiCl stress. Monocultures of WT cells, or cells with the indicated mutants integrated into the HCM1 genomic locus, were grown in control medium or LiCl medium for 40 hours. Cells were then arrested in G1 with alpha-factor for 3 hours, released from the alpha-factor arrest, and cell cycle position was followed by flow cytometry. LiCl was kept in the medium in the indicated samples throughout the experiment. Representative plots for WT cells are shown on the left, quantitation of progression through the cell cycle is shown on the right. An average of n = 3 experiments is shown, and error bars represent standard deviations. Note that all strains growing in LiCl display a similar asynchronous release and are not significantly different from one another (as determined by two-way ANOVA with Geisser-Greenhouse correction and a Dunnett’s multiple comparisons test). (B) Representative FACS plots showing the DNA content of the indicated Hcm1 strains after 40 hours of growth in control or LiCl containing media (left) and percentage of S-phase cells (right). Percentage of S-phase cells is an average of n = 3 biological replicates, error bars represent standard deviations. One-way ANOVA with the Geisser-Greenhouse correction and Tukey’s multiple comparison test showed that none of the samples were significantly different from each other (ns). (TIFF) [file pgen.1011874.s005.tiff]
