# Supplementary material for: Dynamic phosphorylation of Hcm1 promotes fitness in chronic stress
Source: PLoS Genet. 2025 Sep 15;21(9):e1011874. doi: 10.1371/journal.pgen.1011874 (PMC12453243; doi:10.1371/journal.pgen.1011874)
Supplement: S1 Table — All S. cerevisiae strains are in the BY4741 background. (PDF) [file pgen.1011874.s006.pdf]

S1 Table. Strain table

Strain name	Genotype	Figure
YBL192	*MATa his3Δ1 ura3Δ0 leu2Δ0 met15Δ0 HCM1-3V5-KanMX*	1B
YMC50	*MATa his3Δ1 ura3Δ0 leu2Δ0 lys2Δ0 HIS3MX6-GAL1p-HCM1-3HA-KanMX ChrVIΔ181901-182001::Hyg-TEFp-GFP + pRS316-HCM1p-HCM1-3V5*	1C-D, 6C-G, 7D, S1D-E, S4, S5
YMC55	*MATa his3Δ1 ura3Δ0 leu2Δ0 lys2Δ0 HIS3MX6-GAL1p-HCM1-3HA-KanMX ChrVIΔ181901-182001::Hyg-TEFp-GFP(Y66F) + pRS316-HCM1p-hcm1-8E-3V5*	1C-D, 5C, S1D-E, S5
YMC9	*MATa his3Δ1 ura3Δ0 leu2Δ0 lys2Δ0 HIS3MX6-GAL1p-HCM1-3HA-KanMX*	2, 3, 4
YMC625	*MATa his3Δ1 ura3Δ0 leu2Δ0 lys2Δ0 ChrVIΔ181901-182002::Hyg-TEFp-GFP(Y66F) HIS3-GAL1p-HCM1-3HA-KanMx + pRS316-HCM1-3V5*	5B-D
YMC611	*MATa his3Δ1 ura3Δ0 leu2Δ0 lys2Δ0 HIS3-GAL1p-HCM1-3HA-KanMx ChrVIΔ181901-182001::HYG-TEFp-GFP + pRS316-hcm1-3S-3V5*	5B-D
YMC53	*MATa his3Δ1 ura3Δ0 leu2Δ0 lys2Δ0 HIS3MX6-GAL1p-HCM1-3HA-KanMX ChrVIΔ181901-182001::Hyg-TEFp-GFP(Y66F) + pRS316-HCM1p-HCM1-3V5*	6C-E
YMC443	*MATa his3Δ1 ura3Δ0 leu2Δ0 lys2Δ0 HIS3MX6-GAL1p-HCM1-3HA-KanMX ChrVIΔ181901-182001::Hyg-TEFp-GFP + pRS316-HCM1p-hcm1-3N-3V5*	6C-E, 7D
YMC446	*MATa his3Δ1 ura3Δ0 leu2Δ0 lys2Δ0 HIS3MX6-GAL1p-HCM1-3HA-KanMX ChrVIΔ181901-182001::Hyg-TEFp-GFP(Y66F) + pRS316-HCM1p-hcm1-3N-3V5*	6C-G
YMC445	*MATa his3Δ1 ura3Δ0 leu2Δ0 lys2Δ0 HIS3MX6-GAL1p-HCM1-3HA-KanMX ChrVIΔ181901-182001::Hyg-TEFp-GFP + pRS316-HCM1p-hcm1-3N8E-3V5*	6C, 6F-G, 7D
YMC448	*MATa his3Δ1 ura3Δ0 leu2Δ0 lys2Δ0 HIS3MX6-GAL1p-HCM1-3HA-KanMX ChrVIΔ181901-182001::Hyg-TEFp-GFP(Y66F) + pRS316-HCM1p-hcm1-3N8E-3V5*	6C, 6F-G
YMC52	*MATa his3Δ1 ura3Δ0 leu2Δ0 lys2Δ0 HIS3MX6-GAL1p-HCM1-3HA-KanMX ChrVIΔ181901-182001::Hyg-TEFp-GFP + pRS316- HCM1p-hcm1-8E-3V5*	7D, S5
YAR46	*MATa ura3Δ0 his3Δ1 leu2Δ0 lysΔ0 hcm1Δ::KanMX ChrVIΔ181901-2001::HYG-Hcm2BS-GAL1p-GFP + pRS316*	6B, 7A-C, S4
YAR47	*MATa ura3Δ0 his3Δ1 leu2Δ0 lysΔ0 hcm1Δ::KanMX ChrVIΔ181901-2001::HYG-Hcm2BS-GAL1p-GFP + pRS316- HCM1p-HCM1-3V5*	6B, 7A-C, S4
YAR49	*MATa ura3Δ0 his3Δ1 leu2Δ0 lysΔ0 hcm1Δ::KanMX ChrVIΔ181901-2001::HYG-Hcm2BS-GAL1p-GFP + pRS316- HCM1p-hcm1-8E-3V5*	6B, 7A-C, S4
YAR50	*MATa ura3Δ0 his3Δ1 leu2Δ0 lysΔ0 hcm1Δ::KanMX ChrVIΔ181901-2001::HYG-Hcm2BS-GAL1p-GFP + pRS316- HCM1p-hcm1-3N-3V5*	6B, 7A-C, S4
YAR51	*MATa ura3Δ0 his3Δ1 leu2Δ0 lysΔ0 hcm1Δ::KanMX ChrVIΔ181901-2001::HYG-Hcm2BS-GAL1p-GFP + pRS316- HCM1p-hcm1-3N8E-3V5*	6B, 7A-C, S4
YMF448	*MATa his3Δ1 ura3Δ0 leu2Δ0 met15Δ0 ChrVI181901-182001::NAT-TEFp-Crz114-424-GFP-HIS3Mx*	S3
YMF449	*MATa his3Δ1 ura3Δ0 leu2Δ0 met15Δ0 cnb1Δ::KanMx ChrVI181901-182001::NAT-TEFp-Crz114-424-GFP-HIS3Mx*	S3
