# Supplementary material for: Dynamic phosphorylation of Hcm1 promotes fitness in chronic stress
Source: PLoS Genet. 2025 Sep 15;21(9):e1011874. doi: 10.1371/journal.pgen.1011874 (PMC12453243; doi:10.1371/journal.pgen.1011874)
Supplement: S2 Table — Hcm1 expression plasmids. (PDF) [file pgen.1011874.s007.pdf]

S2 Table. Plasmid table

**Plasmid name**	**Description**
pRS316-HCM1-3V5	*HCM1p-HCM1-3V5,* CEN, URA3
pRS316-hcm1-8E-3V5	*HCM1p-hcm1-8E-3V5,* CEN, URA3
pRS316-hcm1-3N-3V5	*HCM1p-hcm1-3N-3V5,* CEN, URA3
pRS316-hcm1-3N8E-3V5	*HCM1p-hcm1-3N8E-3V5,* CEN, URA3
pRS316-hcm1-A/E-3V5	*HCM1p-hcm1-A/E-3V5, CEN, URA3*
pRS316-hcm1-WT/A-3V5	*HCM1p-hcm1-WT/A, CEN, URA3*
pRS316-hcm1-WT/E-3V5	*HCM1p-hcm1-WT/E, CEN, URA3*
pRS316-hcm1-3S-3V5	*HCM1p-hcm1-3S-3V5,* CEN, URA3
