# Supplementary material for: Dynamic phosphorylation of Hcm1 promotes fitness in chronic stress
Source: PLoS Genet. 2025 Sep 15;21(9):e1011874. doi: 10.1371/journal.pgen.1011874 (PMC12453243; doi:10.1371/journal.pgen.1011874)
Supplement: S3 Table — Sequences of oligonucleotides used for Phosphosite Scanning sequencing library construction. (PDF) [file pgen.1011874.s008.pdf]

S3 Table. Oligonucleotide table

Name	TruSeq index number	TruSeq index sequence	Orientation	Sequence
MC71	Universal	N/A	FWD	AATGATACGGCGACCACCGAGATCTACACT CTTTCCCTACACGACGCTCTTCCGATCTCTC ATGGTTCGGACTTACTT
MC72	1	ATCACG	REV	CAAGCAGAAGACGGCATACGAGATCGTGAT GTGACTGGAGTTCAGACGTGTGCTCTTCCG ATCTGGGTGCAGAGGACTTTCT
MC73	2	CGATGT	REV	CAAGCAGAAGACGGCATACGAGATACATCG GTGACTGGAGTTCAGACGTGTGCTCTTCCG ATCTGGGTGCAGAGGACTTTCT
MC80	3	TTAGGC	REV	CAAGCAGAAGACGGCATACGAGATGCCTAA GTGACTGGAGTTCAGACGTGTGCTCTTCCG ATCTGGGTGCAGAGGACTTTCT
MC81	4	TGACCA	REV	CAAGCAGAAGACGGCATACGAGATTGGTCA GTGACTGGAGTTCAGACGTGTGCTCTTCCG ATCTGGGTGCAGAGGACTTTCT
MC82	5	ACAGTG	REV	CAAGCAGAAGACGGCATACGAGATCACTGT GTGACTGGAGTTCAGACGTGTGCTCTTCCG ATCTGGGTGCAGAGGACTTTCT
MC83	6	GCCAAT	REV	CAAGCAGAAGACGGCATACGAGATATTGGC GTGACTGGAGTTCAGACGTGTGCTCTTCCG ATCTGGGTGCAGAGGACTTTCT
MC84	7	CAGATC	REV	CAAGCAGAAGACGGCATACGAGATGATCTG GTGACTGGAGTTCAGACGTGTGCTCTTCCG ATCTGGGTGCAGAGGACTTTCT
MC85	8	ACTTGA	REV	CAAGCAGAAGACGGCATACGAGATTCAAGT GTGACTGGAGTTCAGACGTGTGCTCTTCCG ATCTGGGTGCAGAGGACTTTCT
MC86	9	GATCAG	REV	CAAGCAGAAGACGGCATACGAGATCTGATC GTGACTGGAGTTCAGACGTGTGCTCTTCCG ATCTGGGTGCAGAGGACTTTCT
MC87	10	TAGCTT	REV	CAAGCAGAAGACGGCATACGAGATAAGCTA GTGACTGGAGTTCAGACGTGTGCTCTTCCG ATCTGGGTGCAGAGGACTTTCT
MC88	11	GGCTAC	REV	CAAGCAGAAGACGGCATACGAGATGTAGCC GTGACTGGAGTTCAGACGTGTGCTCTTCCG ATCTGGGTGCAGAGGACTTTCT
MC89	12	CTTGTA	REV	CAAGCAGAAGACGGCATACGAGATTACAAG GTGACTGGAGTTCAGACGTGTGCTCTTCCG ATCTGGGTGCAGAGGACTTTCT
MC90	14	AGTTCC	REV	CAAGCAGAAGACGGCATACGAGATGGAACT GTGACTGGAGTTCAGACGTGTGCTCTTCCG ATCTGGGTGCAGAGGACTTTCT
MC91	15	ATGTCA	REV	CAAGCAGAAGACGGCATACGAGATTGACAT GTGACTGGAGTTCAGACGTGTGCTCTTCCG ATCTGGGTGCAGAGGACTTTCT
MC92	16	CCGTCC	REV	CAAGCAGAAGACGGCATACGAGATGGACG GGTGACTGGAGTTCAGACGTGTGCTCTTCC GATCTGGGTGCAGAGGACTTTCT

MC93	19	GTGAAA	REV	CAAGCAGAAGACGGCATACGAGATTTTCAC GTGACTGGAGTTCAGACGTGTGCTCTTCCG ATCTGGGTGCAGAGGACTTTCT
MC94	20	GTGGCC	REV	CAAGCAGAAGACGGCATACGAGATGGCCAC GTGACTGGAGTTCAGACGTGTGCTCTTCCG ATCTGGGTGCAGAGGACTTTCT
MC95	21	GTTTCG	REV	CAAGCAGAAGACGGCATACGAGATCGAAAC GTGACTGGAGTTCAGACGTGTGCTCTTCCG ATCTGGGTGCAGAGGACTTTCT
MC96	22	CGTACG	REV	CAAGCAGAAGACGGCATACGAGATCGTACG GTGACTGGAGTTCAGACGTGTGCTCTTCCG ATCTGGGTGCAGAGGACTTTCT
MC97	23	GAGTGG	REV	CAAGCAGAAGACGGCATACGAGATCCACTC GTGACTGGAGTTCAGACGTGTGCTCTTCCG ATCTGGGTGCAGAGGACTTTCT
MC98	25	ACTGAT	REV	CAAGCAGAAGACGGCATACGAGATATCAGT GTGACTGGAGTTCAGACGTGTGCTCTTCCG ATCTGGGTGCAGAGGACTTTCT
MC99	27	ATTCCT	REV	CAAGCAGAAGACGGCATACGAGATAGGAAT GTGACTGGAGTTCAGACGTGTGCTCTTCCG ATCTGGGTGCAGAGGACTTTCT
MC112	13	AGTCAA	REV	CAAGCAGAAGACGGCATACGAGATTTGACT GTGACTGGAGTTCAGACGTGTGCTCTTCCG ATCTGGGTGCAGAGGACTTTCT
MC113	18	GTCCGC	REV	CAAGCAGAAGACGGCATACGAGATGCGGAC GTGACTGGAGTTCAGACGTGTGCTCTTCCG ATCTGGGTGCAGAGGACTTTCT
MC114	17	GTAGAG	REV	CAAGCAGAAGACGGCATACGAGATCTCTAC GTGACTGGAGTTCAGACGTGTGCTCTTCCG ATCTGGGTGCAGAGGACTTTCT
MC155	28	CAAAAG	REV	CAAGCAGAAGACGGCATACGAGATCTTTTG GTGACTGGAGTTCAGACGTGTGCTCTTCCG ATCTGGGTGCAGAGGACTTTCT
MC156	29	CAACTA	REV	CAAGCAGAAGACGGCATACGAGATTAGTTG GTGACTGGAGTTCAGACGTGTGCTCTTCCG ATCTGGGTGCAGAGGACTTTCT
MC157	30	CACCGG	REV	CAAGCAGAAGACGGCATACGAGATCCGGTG GTGACTGGAGTTCAGACGTGTGCTCTTCCG ATCTGGGTGCAGAGGACTTTCT
HCM1-PMF	N/A	N/A	FWD	CCTTCCTCTCATGGTTCGGA
MC70	N/A	N/A	REV	CAAGGGATTGGGGATTCCAT
