# Supplementary material for: Dynamic phosphorylation of Hcm1 promotes fitness in chronic stress
Source: PLoS Genet. 2025 Sep 15;21(9):e1011874. doi: 10.1371/journal.pgen.1011874 (PMC12453243; doi:10.1371/journal.pgen.1011874)
Supplement: Attachment — Submitted filename: Conti_Response.pdf [file pgen.1011874.s014.pdf]

Response to Editorial and Reviewer Comments

Additional Editor Comments:
Your manuscript was reviewed by two experts in the field. Both reviewers expressed concerns that there are insufficient evidence to support the role of Cks1-dependent priming sites and the pulsatile nature of Hcm1 activation. Reviewer 1 suggested performing phosphosite scanning experiments using TP-to-SP mutants, which would permit phosphorylation while preventing Cks1 binding. To assess whether calcineurin-mediated regulation of Hcm1 enhances stress fitness, they also proposed generating an Hcm1 PxIxIT motif mutant to test for fitness loss under stress conditions. Reviewer 2 also noted that some conclusions are indirect and not fully supported, particularly regarding Cks1-dependent priming and Hcm1 pulse phosphorylation. Both reviewers also raised several minor concerns. Therefore, we expect authors to resubmit the manuscript after "major revision" in 60 days (an extension can be granted if needed).

We thank the editors and reviewers for their thoughtful feedback. In response, we have conducted additional experiments and revised the manuscript to clarify our conclusions, particularly regarding Cks1-dependent priming and the dynamic regulation of Hcm1. We have added new data and analyses that reinforce the importance of dynamic phosphorylation in promoting stress fitness. While some mechanistic aspects remain difficult to demonstrate directly, we have taken care to present our conclusions with appropriate caution. We also restructured the manuscript for clarity, addressed all minor comments, and corrected typographical errors. We hope these revisions meet the expectations for resubmission.

Reviewer #1: In this report, Conti et al. investigate how dynamic phosphorylation of a yeast transcription factor, Hcm1, provides a fitness advantage under chronic stress conditions. They show that Hcm1 is rapidly dephosphorylated in response to LiCl stress and that constitutive activation by phosphomimetic mutations imparts a fitness disadvantage. They use phosphosite scanning to assess how phospho-null and phosphomimetic mutations in the Hcm1 transactivation domain (TAD) affect cellular fitness. This shows that nearly all mutations impart a reduced fitness advantage under stress conditions relative to non-stress controls. The authors attribute this to the disruption of phosphorylation dynamics, as opposed to simple changes in total activity or phosphorylation levels. To support this model, they use stabilizing mutations to show that increasing Hcm1 activity can provide a fitness advantage under all conditions, but only when TAD phosphorylation can also be dynamically regulated. Phosphosite scanning also allowed them to identify two putative CKS1 priming sites, in addition to the two that have already been established. Beyond the effects on fitness, they show that mutations that prevent dynamic phosphorylation disrupt Hcm1 transcriptional outputs during chronic stress.
This work provides compelling evidence that dynamic phosphorylation regulates Hcm1 signaling and is necessary for optimal fitness under stress. However, the narrative is difficult to follow, and we suggest it be restructured before publication. Additionally, this work would be substantially strengthened with experimental evidence to tie the effects on fitness they observe to the molecular mechanisms they describe. Overall, this work addresses underappreciated and difficult to study component of signaling networks. It represents a significant step forward in the field and we support its publication with the following comments and suggestions:

We thank the reviewer for recognizing the significance of our study. We have restructured portions of the text and added additional experiments to strengthen the conclusions, as described below.

1. The EE mutation is described as a separation of function, isolating CKS1 dependent effects from all others. However, phosphomimetic mutations may not fully recapitulate other components of phosphorylation function. Phosphosite scanning experiments using TP to SP mutants, which would facilitate phosphorylation while still preventing CKS1 binding, would help to show defects in CKS1 priming are responsible for the observed reduction in fitness.

We agree that a further examination of potential priming sites would be a good addition to our paper. For reasons explained below, we have not performed a Phosphosite Scanning screen to investigate all combinations of TP to SP mutations but have examined many rationally designed mutants to address this issue. These data are included below in **Fig R1**, and in the revised **Fig 5B-D**.

The mutants that we have analyzed are diagrammed in **Fig R1A**. In our previous publication (Conti et al, *Nat Commun*, 2023, PMID: 36658165), we characterized the 4S, 3S and 1S mutants, which disrupt Cks1 priming sites in the N-terminal half of the TAD (here, called group 1 sites). We found that mutation of the first 3 or 4 sites in the TAD from TP to SP (in the 4S and 3S mutants) significantly reduced phosphorylation of Hcm1. Mutation of the 4th site alone (the 1S mutant) had little to no effect. These data support the conclusion that the first 3 sites (T428, T440, T447) are Cks1 priming sites. The previously published result is repeated below in **Fig R1B**.

In the current study, we found that phosphomimetic mutations at group 2 sites (T479 and T486; **Fig R1A**) had a negative impact on fitness when combined with WT sites in the WT/E library, specifically in stress (new **Fig 4E and 5A**). This suggests that T479 and T486 might also be Cks1 priming sites, and that priming via these sites is of increased importance in stress. To test this, we made SP mutations at these sites and examined their phosphorylation in cells. Mutation of just these two TPs to SPs (the 2S mutant) did not reduce phosphorylation compared to WT (**Fig R1B**). Surprisingly, we also found that when the 2S mutant was combined with the previously characterized 4S mutant—thereby removing all potential Cks1 priming sites— the resulting 6S mutant displayed greater phosphorylation than the 4S mutant.

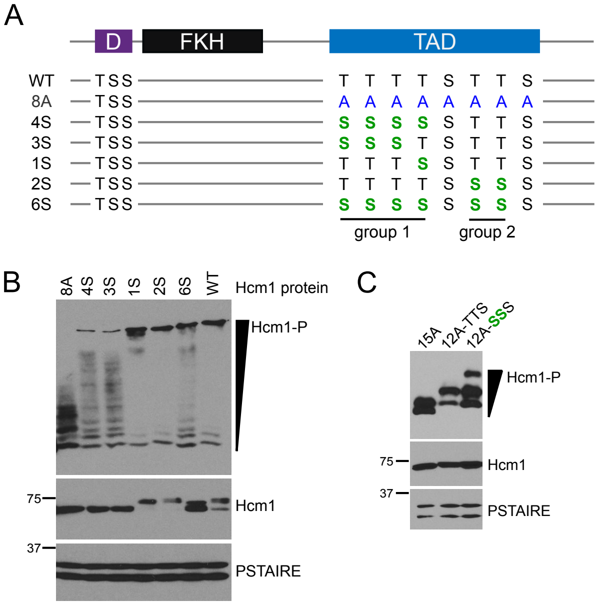

Fig R1. Analysis of SP mutants. (A) Diagram of TP to SP mutants that are included in part (B) and described below. (B) Western blot of the V5 tag on Hcm1, from a phostag gel that allows comparison of the phosphorylation state of the indicated Hcm1 mutants. Also shown is a V5 Western blot of a standard SDS-PAGE, which has less resolution and makes it difficult to compare phosphosite mutants. PSTAIRE is shown as a loading control. (C) Phostag and standard Western blots of the indicated Hcm1 mutants, as in (B).

While this result seems counterintuitive, previous work from the Loog lab has demonstrated that serines are phosphorylated more efficiently than threonines by CDK (Ord et al, *Nat Struct Mol Biol*, 2019, PMID: 31270471). Therefore, we considered the possibility that mutation of T479 and T486 to SP resulted in increased phosphorylation at those sites, potentially obscuring any decreased phosphorylation at the downstream site S496 due to lack of Cks1 priming. To test this directly we introduced specific phosphosites into the backbone of the Hcm1-15A mutant, which has all S/T-P sites in the protein mutated to A-P (Landry et al, *EMBO*, 2014, PMID: 24714560), in order to provide greater resolution on a phostag gel. We added back the last three S/T-P sites (T479, T486, S496) to the 15A mutant to construct the 12A-TTS mutant. Phosphorylation of these C-terminal sites can be observed by comparing the 15A mutant to the 12A-TTS mutant on a phostag gel (**Fig R1C**). We then

mutated the two potential Cks1 priming sites (T479 T486) to generate the 12A-SSS mutant. As shown in **Fig R1C**, the 12A-SSS mutant shows increased phosphorylation compared to the 12A-TTS mutant, confirming that serines at positions 479 and 486 sites undergo increased phosphorylation. For this reason, we cannot determine whether they also disrupt Cks1-dependent priming.

Because of this complication, we have not performed Phosphosite Scanning of all possible TP to SP combinations. In short, since T to S mutations impair Cks1-dependent priming, but enhance phosphorylation by CDK, the results of such a screen would not be interpretable.

We also did not perform additional experiments with the group 2 site mutants, since it was not possible to provide additional evidence that T479 and T486 function as priming sites *in vivo*. However, we did perform additional experiments with the 3S mutant, which disrupts group 1 priming sites. To test whether Cks1-dependent priming is important for fitness in stress, we performed pairwise fitness assays of *hcm1-3S* mutants and WT cells (see new **Fig 5B-D**). Notably, we found that the 3S mutant showed reduced fitness in control conditions, and that there was a similar reduction in fitness in stress. This result is similar to what is observed for other mutants that have greatly reduced or no phosphorylation (see mutants with 0-2 phosphomimetic substitutions in **Fig 2D**). Mutants of this type display similarly reduced fitness in both control and stress conditions. These new fitness experiments with the 3S mutant highlight the importance of Cks1-dependent priming in both environments.

2. This story focuses on phosphorylation dynamics but does not directly address the phosphatase, calcineurin. If calcineurin is the only phosphatase targeting Hcm1, preventing interactions between it and Hcm1 (by mutating the Hcm1 PxIxIT motif) would result in constitutively hyper-phosphorylated Hcm1. If dynamic phosphorylation is required for optimal fitness, then preventing calcineurin-mediated regulation should result in a fitness loss under stress when compared to non-stress conditions.

We thank the reviewer for the suggestion and agree that, in principle, this is a good experiment. We previously constructed and characterized an Hcm1 mutant with a deletion in the CN docking motif (ΔPSIEIQ, see Arsenault et al., *Mol Biol Cell*, 2015, PMID: 26269584). However, one important consideration is that while this mutation reduces dephosphorylation of Hcm1 by CN, it does not completely block it. Our previous work showed that Hcm1-ΔPSIEIQ still undergoes significant dephosphorylation by CN *in vitro* and partial dephosphorylation *in vivo* in response to LiCl. Similar results are observed with numerous other CN substrates: deletion of the PxIxIT motif reduces but doesn't prevent dephosphorylation of many substrates by CN (see Goldman et al, *Mol Cell*, 2014, PMID: 24930733). This complicates the interpretation of the proposed experiment.

Nonetheless, we did perform the requested experiment (**Fig R2**). We confirmed that the ΔPSIEIQ mutant was expressed at a similar level as WT Hcm1 (**Fig R2A**) and examined its fitness in pairwise competition assays in control and stress conditions (**Fig R2B-C**). Unfortunately, we did not observe any fitness defect in the mutant, in either condition. For the reasons mentioned above, this could be because CN can still dephosphorylate the mutant protein, albeit to a lesser extent. Given the limitations in interpreting these results, we have opted not to include the data in the revised manuscript.

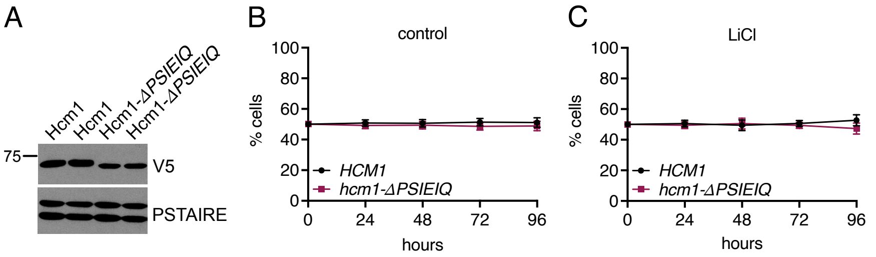

Fig R2. Deletion of the CN docking motif in Hcm1 does not impact fitness. (A) Western blot of WT and ΔPSIEIQ proteins. (B-C) Pairwise competition assays in control and LiCl conditions. Shown is an average of n=6 replicates.

Since we were aware of this issue at the start of this project, we focused on the 8E mutant throughout the manuscript. The 8E mutant represents the cleanest approach to examine a constitutively "phosphorylated" protein, and since it loses its fitness advantage in stress, we conclude that dephosphorylation is important. We cannot rule out the possibility that an additional phosphatase, in combination with CN, contributes to this regulation.

3. Figure S3B shows Hcm1-8E and Hcm1-3N8E have reduced expression under LiCl treatment. Why is this the case and could it intersect the effects on survival the author's report?

It is likely that expression of the 8E and 3N8E proteins are expressed at slightly lower levels in LiCl in the reporter experiments (new **S4B Fig**) because both of those populations have an increase in the fraction of cells in G1, compared to the other mutants in LiCl (see **S4C Fig**). Since the mutants are all expressed from the endogenous *HCM1* promoter, and *HCM1* is not transcribed in G1 cells, it is possible that fewer cells express Hcm1. This cell cycle skewing probably occurs because 8E and 3N8E cells are not as fit as the other strains in stress and are dividing slower. It is possible that this could contribute to the reduction in reporter gene expression in the pooled population, which is why we have chosen to instead emphasize the finding that the activity of WT and Hcm1-3N proteins increase in stress, in support of our model.

4. In figure 1D, it's not clear that the growth disadvantage brought on by hcm1-8e is consistent over time. Why is this the case? Do cells adapt to higher activity over time?

We agree that the growth disadvantage of the *hcm1-8E* mutant does not appear consistent over time. We often observe this with mutants that have modest fitness phenotypes. To examine this data more carefully, we performed a statistical analysis and found that, although a trend towards a fitness disadvantage in the *hcm1-8E* mutant in LiCl is observed, there is no statistically significant difference between the percentage of *hcm1-8E* cells at each time point (see **S4 Dataset - Fig 1C**). In contrast, there is a significant increase in *hcm1-8E* cells at each time point when cells are growing in control conditions (see **S4 Dataset - Fig 1D**). We have reworded the text to include this information and to emphasize the conclusion that the fitness benefit provided by Hcm1-8E is lost in stress.

5. Beginning at line 116, it is stated that cells were diluted every 24 hours to allow them to exit the cell cycle. Why is this a benefit?

This use of 24-hour time points is a benefit for practical reasons—it is technically easier to perform the experiments when cells are diluted every 24 hours instead of every 12 hours. The point we were trying to make with this statement is that although this dilution protocol does allow cells to exit the cell cycle as they become saturated, it doesn't impact the results. We have reworded this section to clarify the meaning (see lines 131-137).

6. In the third paragraph of the introduction, beginning at line 36, the authors write that "stressors signal" and "coordinate the stress response program". This reads as though the stressors exhibit control over the cell as opposed to the cell responding to the stressor, which is likely more appropriate.

We have reworded this sentence, as suggested.

7. Figure panel ordering is inconsistent and hard to follow. In particular, figure 4 needs attention.

Figure panels have been reordered for clarity.

8. The title of the section on line 244 "Frequency modulation enhances Hcm1 activity in stress" does

not seem appropriate. The associated experiments do not address how signaling amplitude or frequency impact Hcm1 signaling.

We agree and have changed the section heading as suggested.

9. Figures S1B and S1C do not appear to be labeled correctly. Both grey and black points are labeled only as HCM1.

We apologize for the lack of clarity in the labeling. Both strains do express WT Hcm1, but they differ in their GFP markers. We have added this information to the labels.

10. Throughout the text, there are small typos that need attention. For example, on line 318, "Csk1" should be corrected to "Cks1." Additionally, it would be helpful to maintain consistency with phosphorylation site nomenclature by consistently using either "T" (Threonine) or "S" (Serine) in front of the residue number.

We have made the suggested edits.

Reviewer #2: Review of "Dynamic phosphorylation of Hcm1 promotes fitness . . . ", by Conti . . . Benanti, for PLoS Genetics

The authors make various mutations in the HCM1 gene that interfere with phospho-regulation of the protein, and measure the fitness of these mutants under normal and stressed conditions. They find that phospho-regulation contributes to fitness.

I think the main issue with the manuscript is that it wasn't that interesting. I felt it was a long hard read, with small effects, and in the end, I didn't learn that much.

PLoS Genetics lists some criteria for publication, and I'll go through these.

Originality. The work is reasonably original. Not highly original—mutating phosphorylation sites and assaying phenotypes is a standard thing. But there are original aspects here, doing it with Hcm1 and in many combinations and measuring fitness.
High importance to researchers in the field. My feeling is no, it isn't that important to workers in the field. The eventual conclusion is more-or-less what one would have said, that phosphorylation is for dynamic control.
Broad interest. Again, my feeling is no. This is not really a topic of broad interest.
Rigorous methodology. Yes. A tremendous amount of good, hard work went into this manuscript, there are a lot of data, and the data look very reliable.
Substantial evidence for its conclusions. Yes and no. The data are very good, and they show what they show. On the other hand, some of the conclusions—the inferences—are indirect, and are not conclusive. I am thinking for instance of the inferences about priming with Cks1, and about the need for pulses of Hcm1 phosphorylation. I thought the arguments for these inferences were reasonable, but still far from conclusive. "Substantial", I'm not sure.

We thank the reviewer for recognizing the originality and rigor of our study. We have addressed their concerns below and in the revised manuscript, to better convey the importance of the work and provide better support for our conclusions.

Some scientific points:

The disadvantage of hcm1-8E cells in stress. Lines 92 to 94 say that hcm1-8E cells have a fitness defect in stress (Fig. 1D). This finding is sort of what the paper is all about. But then, to the contrary, lines 131 to 133, the same mutant “had a slight fitness advantage in pooled screens (Fig. 2D . . .” So, the thing the paper is about—the mutant’s fitness disadvantage—isn’t even true when the authors do the assay a different way. To their credit, they recognize this and point it out, but even so, it’s an issue. Of this issue, they write: “This observation is consistent with previous findings that pooled screens result in higher selection coefficients than pairwise competition assays and is likely due to technical differences in the experimental approach . . .” Well, ok, but this doesn’t really explain anything to me, or make me feel better about the opposite results.

As described in response to R1, point 4, above, the fitness defect observed with the 8E mutant in stress is modest, and although there is a clear trend towards a fitness deficit, our new analysis shows the percentage of 8E cells is not significantly changing over time. However, what is clear is that the large fitness benefit provided by the 8E mutant in control conditions is completely lost in stress. In control conditions the percentage of 8E cells significantly increases at each time point.

It is true that we see a slight fitness advantage is retained in this mutant when we perform a pooled screen instead of a pairwise assay. There could be many reasons for this, but it is not surprising that the results differ slightly when a strain is competing with one other strain vs hundreds of different strains. The point we'd like to emphasize is that there is significant reduction in fitness in stress, relative to wild type, in both assays. In other words, phosphomimetic mutations no longer provide the same fitness advantage in stress, which is consistent with our model that dynamic removal and addition of phosphates is necessary to stimulate Hcm1 activity in stress.

Also on this subject, I have to wonder a bit about the math of the selection coefficient, which is what they’re measuring in these assays. In a pairwise comparison, the math is straightforward, and they describe it in Methods, and that is fine. But in a pooled comparison, it seems to me more complicated, and I didn’t see a detailed description of what calculation they actually perform. So, for instance, suppose we have a pairwise comparison of strains A and B. Selection coefficient is straightforward. But now suppose we have A, B, and C (the pooled strategy). Suppose C does best and expands in the population, B does second best, but contracts, and A does worst and contracts a lot. Now, by the usual convention, B has a negative selection coefficient. But, it does better than A. So what are they reporting here? The absolute coefficient? I guess not, but they don’t say. Or the relative coefficient, relative to WT? I think so. But now I’m not 100% sure that the math is still the obvious math.

We apologize for not being clear on how selection coefficients were calculated in our screens. In all pooled screens we included WT in the pool, and all selection coefficients were calculated relative to WT (WT has a selection coefficient of zero). This is an important point, because in stress all strains become less fit (see **Fig 7B-C** and compare the total number of generations in all strains between control and stress conditions). Therefore, when we observe a reduced selection coefficient for a particular mutant in stress, that means it displays a greater loss of fitness than a WT strain does. We have edited the results section and the methods to make sure this point is clear.

B. Cks1 priming. There was a lot of text about Cks 1 priming. On lines 174-175, they write “. . . which is consistent with Hcm1 regulation by Cks.” And that is right, it is consistent. But being “consistent” is a long, long way from proof that there is an important Cks1 priming effect. They do have other arguments, but all of them seemed to me indirect and inconclusive. And, mostly, the Ser mutations looked a lot like the Thr mutations, maybe arguing (gently) that the effects are not priming effects. So here, I thought the authors were probably right, but it is a long way from conclusive, and that is usually not good enough for publication.

One could ask, what experiments might support a role for Cks1 here? And there are experiments. But they involve doing a lot more long hard work, and I don’t see that ultimately it matters all that much

anyway whether there is Cks1 priming or not, and so I do not want to recommend that any of these experiments be done.

We agree with the reviewer that conclusively demonstrating Cks1 priming is difficult. As described above in response to R1, point 1, we have performed several experiments aimed at providing stronger evidence for Cks1 priming, particularly at the newly identified putative priming sites, T479 and T486. The standard in the field is to change TP sites to SP sites, so they can not bind Cks1. However, as described above, this is complicated by the fact that serines can be phosphorylated more efficiently than threonines by CDK. For this reason, we describe sites T479 and T486 as putative sites and do not make any definitive claims. Even though these data are not conclusive, on their own, we believe they support our model that dynamic phosphorylation is important and are of value to include in the manuscript.

C. Pulses of Hcm1 activity. The authors argue towards the end of Results that pulses of Hcm1 activity are important. The phosphatase calcineurin (CN) is unusual, in that it has rapid (minutes? I forget) cycles of on/off activity, which lead to pulses in the activity of a client transcription factor, Crz1. The authors argue that there might also be similar rapid pulses in activity of Hcm1, and these pulses might be important (and this would be quite a novel thing.). However, first, the authors have no direct evidence for any pulsing in Hcm1 activity. Second, even if there is "pulsing", it could be on the cell-cycle time-scale (which would be an ordinary, non-novel possibility, since Hcm1 is a cell cycle transcription factor) rather than a Crz1-like minutes (?) timescale.

We agree with the reviewer that we do not demonstrate pulsing of Hcm1 activity directly. Unlike Crz1 and several other transcription factors, Hcm1 does not change localization upon dephosphorylation, so there is no way to visualize phosphorylation dynamics in real time. From our proliferation measurements and cell cycle analyses, we estimate that S phase lasts ~30 minutes in LiCl stress. Published data on CN activation suggests it has pulses of activity that last only few minutes each (Cai et al, *Nature*, 2008, PMID: 18818649). Therefore, it is reasonable to infer that Hcm1 undergoes pulses of dephosphorylation, followed by re-phosphorylation, during S-phase.

However, to our knowledge the pulsing of CN activity has only been demonstrated in cells exposed to 200mM $CaCl_2$ and not in other stressors. Therefore, to gain further support for our model, we examined the dynamics of CN activation in LiCl, using a fluorescent CN reporter developed in our lab (Flynn et al, *Mol Biol Cell*, 2024, PMID: 39083354). These data are included in new **S3 Fig**. We show that the reporter is activated by LiCl in WT cells, but not CN mutant (*cnb1Δ*) cells, and that a low level of activation persists throughout a 72-hour time course, similar to what is observed in $CaCl_2$. We also perform live cell imaging and demonstrate that after the initial wave of CN activity subsides, pulses of CN activity lasting < 5 min are observed in individual cells. Therefore, CN activation dynamics are similar between cells exposed to $CaCl_2$ and LiCl, and the pulses of dephosphorylation that Hcm1 is expected to undergo in LiCl are shorter than the duration of S-phase.

Here, it might be nice to do an experiment using a stressor that doesn't work through calcineurin (say, high growth temperature, or an ethanol carbon source), and see if the hcm1 mutations have the same effect as when the stressor works through calcineurin. This gets at the pulsatile nature of calcineurin, and also uses stressors that are maybe more often relevant for yeast.

We thank the reviewer for this suggestion and agree that, in principle, it would be a valuable experiment. However, in our hands, all stressors that we have tested to date activate CN. In regard the stressors the reviewer suggests, both high temperature and ethanol have been reported to activate CN (Bultynck et al, *MCB*, 2006, PMID: 16738335; Araki et al, *J of Bioscience and Bioengineering*, 2009, PMID: 19147100). To confirm this in our system, we tested heat stress at 37°C and observed that it activated the CN reporter to a similar extent as LiCl (**Fig R3**). These findings

suggest that CN-dependent regulation of Hcm1 likely occurs during the response to a broad range of stress conditions.

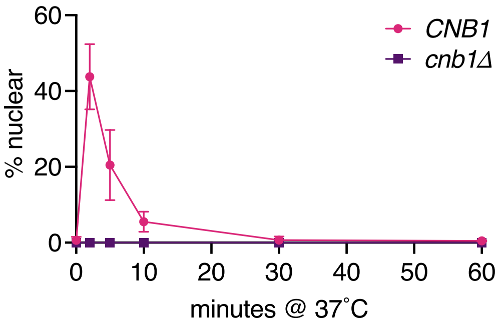

Fig R3. Temperature stress activates CN. Cells expressing a fluorescent CN reporter (CNR) that translocates to the nucleus upon activation. Cells were imaged before and after heat shock at 37ºC. The reporter was activated in WT (*CNB1*), but not CN mutant (*cnb1Δ*) cells. Shown is an average of n=2 replicates.

Ultimately, the author's main conclusion—that dynamic phosphorylation of Hcm1 contributes to fitness under stress—is well-supported and convincing. But it is well-established that, in general, phosphorylation is for situations where dynamic regulation is needed, so it is hard to see this as a big step forward. Also, the "dynamic" phosphorylation could just be garden-variety cell cycle phosphorylation. Some of the other conclusions—about Cks1 priming, and the possibly pulsatile nature of Hcm1 activation—were not so well supported.

We believe the key advance of our study is the demonstration that both phosphorylation and dephosphorylation are required to achieve maximal Hcm1 activity and promote cellular fitness under stress. While direct observation of rapid phosphorylation–dephosphorylation cycles remains technically challenging, our genetic analyses offer strong support for this dynamic regulatory model. Importantly, our findings introduce a novel concept: that transient phosphorylation pulses can activate a transcription factor independently of localization changes. We consider this a significant contribution to the broader understanding of transcription factor regulation.
